# Long non-coding RNAs in non-small cell lung cancer as biomarkers and therapeutic targets

**DOI:** 10.1111/jcmm.12431

**Published:** 2014-10-09

**Authors:** Jing Chen, Rui Wang, Kai Zhang, Long-Bang Chen

**Affiliations:** Department of Medical Oncology, Jinling Hospital, School of Medicine, Nanjing UniversityNanjing, Jiangsu, China

**Keywords:** long non-coding RNA, non-small cell lung cancer, biomarker, molecular target

## Abstract

Lung cancer-associated mortality is the most common cause of cancer death worldwide. Non-coding RNAs (ncRNAs), with no protein-coding ability, have multiple biological roles. Long non-coding RNAs (lncRNAs) are a recently characterized class of ncRNAs that are over 200 nucleotides in length. Many lncRNAs have the ability of facilitating or inhibiting the development and progression of tumours, including non-small cell lung cancer (NSCLC). Because of their fundamental roles in regulating gene expression, along with their involvement in the biological mechanisms underlying tumourigenesis, they are a promising class of tissue- and/or blood-based cancer biomarkers. In this review, we highlight the emerging roles of lncRNAs in NSCLC, and discuss their potential clinical applications as diagnostic and prognostic markers and as therapeutic targets.

- Introduction
- LncRNAs and their functions
- Expression of LncRNAs in NSCLC
- Onco-lncRNAs
- MALAT1
- CCAT2
- HOTAIR and AK126698
- Tumour-suppressor lncRNAs
- MEG3
- GAS6-AS1
- BANCR
- Conclusions and future directions

## Introduction

Lung cancer, comprising non-small cell lung cancer (NSCLC) and small cell lung cancer, is the leading cause of cancer-related death worldwide. Although progress in clinical and experimental oncology have been made in recent years, the prognosis of lung cancer patients is still unsatisfactory and the 5-year overall survival rate of lung cancer is only 11% [1].

Non-small cell lung cancer accounts for ∼85% of all lung cancer cases [2]. Despite advances in understanding the molecular mechanisms underlying NSCLC and improvements in NSCLC treatments, the overall survival time is still limited. Because of the insensitivity of diagnostic techniques, many patients are diagnosed at advanced stages and therefore miss the best time for intervention; early-stage NSCLC patients always suffer from tumour metastasis, despite surgery. Molecular-targeted therapy aimed at patients with lung adenocarcinoma (LAD) has significantly improved the survival rate of NSCLC patients; however, there are still many patients who show no response to treatment. Consequently, less than 20% of patients survive more than 5 years [3]. The molecular mechanisms underlying NSCLC have not been fully elucidated and a greater understanding of the development and progression of NSCLC is essential for making early diagnosis, providing early treatment and achieving a better prognosis.

Investigation into the molecular mechanisms of tumourigenesis has typically focused on protein-coding genes. Recent high-throughput transcriptome analysis has discovered that more than 90% of the transcriptome is transcribed into non-coding RNAs, among which microRNAs (miRNAs) have been identified to be involved in multiple biological processes [4–8], including malignant behaviours in lung cancer. Following the demonstration of miRNA involvement in lung cancer, focus has also been given to another class of non-coding RNA, long non-coding RNAs (lncRNAs). LncRNAs are over 200 nucleotides (nt) in length and have no or limited coding protein capacity. LncRNAs are predicted to modulate chromatin or to function as genetic regulators, which depend on their location to the nucleus [9]. To date, more than 3000 lncRNAs have been identified; however, functions for only 1% of them have been proposed. Although the potential functions of lncRNAs are still enigmatic, some studies have found that they are capable of regulating various cellular processes, such as proliferation, cell growth and apoptosis. The aberrant expression of lncRNAs is linked with tumourigenesis, including the pathogenesis of NSCLC and there is accumulating evidence indicating that lncRNAs are involved in the mechanisms of NSCLC development and progression. Studying the functions of lncRNAs opens new avenues into the biology of NSCLC and provides novel possibilities for developing more efficient treatments.

## LncRNAs and their functions

**Fig. 1:**
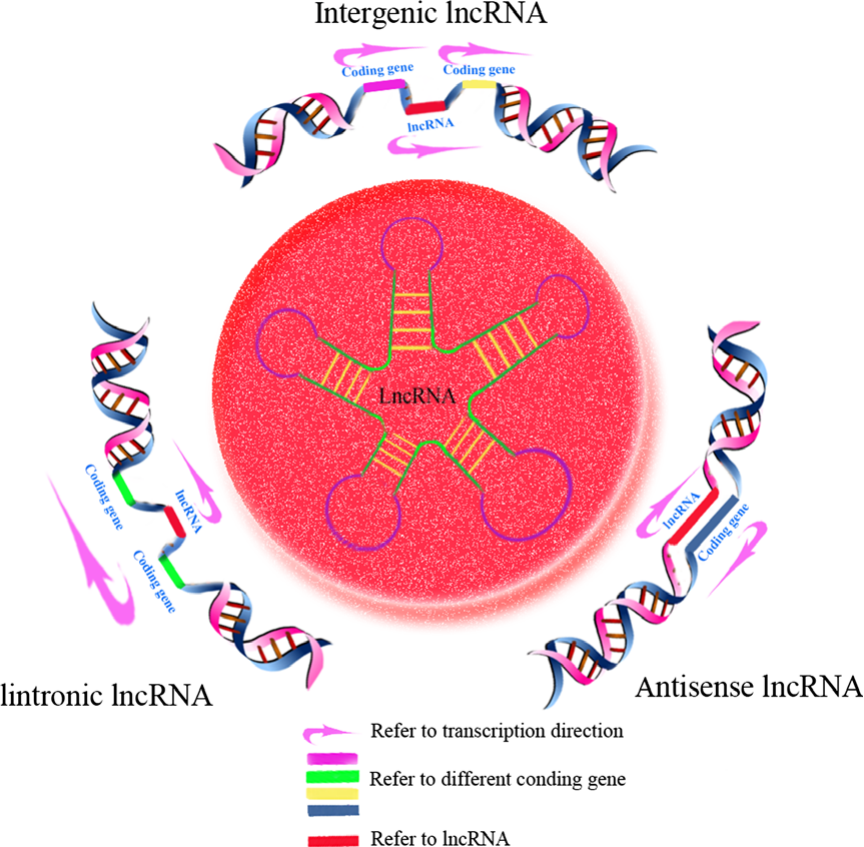
Classification of long non-coding RNAs (lncRNAs) based on location relative to nearby coding genes. Intergenic lncRNAs: transcribed from the regions between two coding genes; intronic lncRNAs: transcribed from the introns of coding genes; antisense lncRNAs: transcribed from the opposite strand of coding genes.

The human genome sequencing projects have revealed that 2% of genes encode proteins, while over 90% of genes encode RNAs that are not translated. [10]. Non-coding RNAs which have limited or no protein-coding capacity can be classified into two types: housekeeping ncRNAs and regulatory ncRNAs. Regulatory ncRNAs are always expressed in a spatial- and/or temporal-specific pattern. According to their size, regulatory ncRNAs can be further divided into two subclasses; small non-coding RNAs whose transcripts are shorter than 200 nt, and lncRNAs that are 200 nt to 100 kilobases (kb). The former contain miRNAs, small nucleolar RNAs, small interfering RNAs (siRNAs), small nuclear RNAs and PIWI-interacting RNAs. The latter are recognized for their versatile roles in transcriptional regulation. The origin of lncRNAs is not very clear. Ponting *et al*. suggested that lncRNAs may arise in five different ways [11]: (*i*) the open reading frame of a protein-coding gene accumulates disruptions and transforms into a functional ncRNA; (*ii*) two separated sequences juxtapose following the rearrangement of a chromosome; (*iii*) there is retrotransposition of a non-coding gene; (*iv*) tandem duplication events create neighbouring repeats within a non-coding RNA; (*v*) there is insertion of a transposable element. Based on genomic location, lncRNAs can be classified into three subtypes (Fig. 1) [12].

**Fig. 2:**
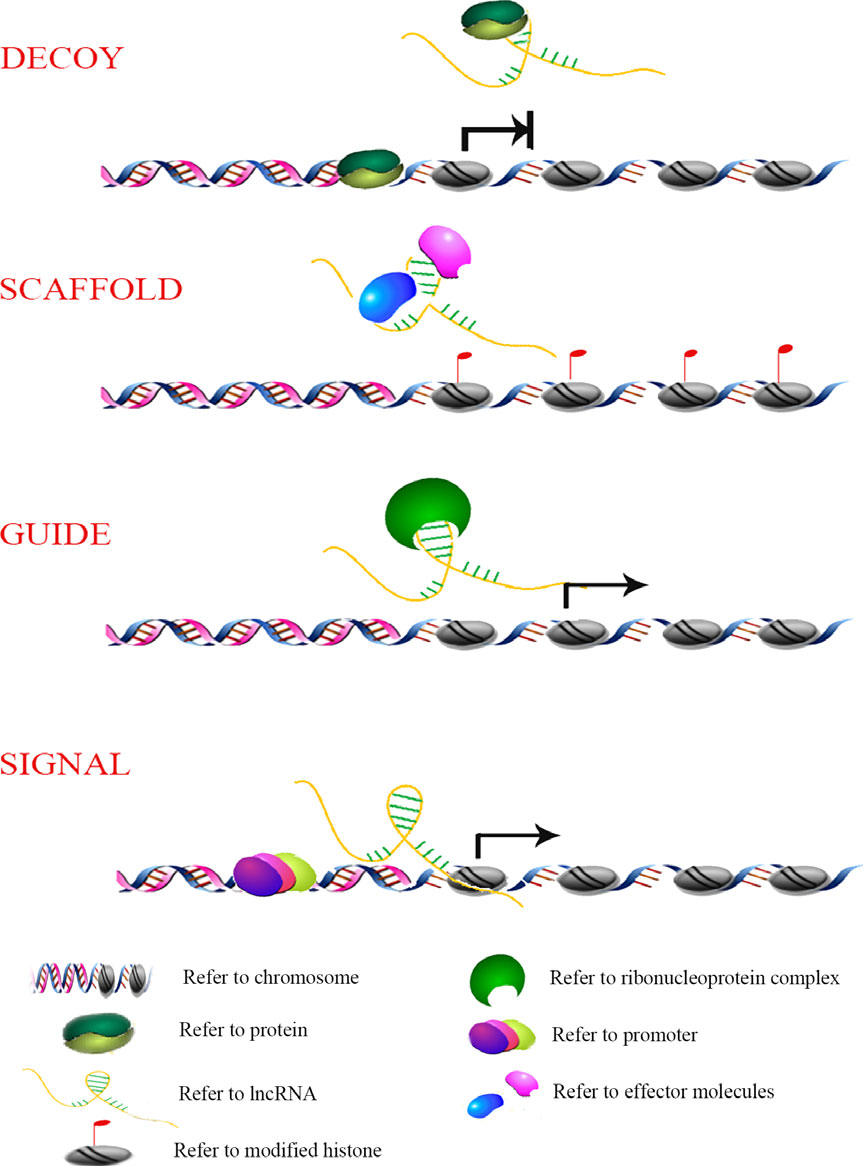
Schematic diagrams of four lncRNA archetypes. Decoys: binding to proteins and titrating them away from chromatin, acting as a ‘molecular sink’; scaffolds: providing a platform to assemble different effector molecules to function together; guides: binding to protein and then directing the localization of the ribonucleoprotein complex to specific target genes to regulate their expression; signals: function as molecular signals to indicate gene regulation in space and time.

LncRNAs are involved in a variety of biological functions. The execution of these functions is based on four archetypes: signals, decoys, guides, scaffolds (Fig. 2) [13]. Signals: these types of lncRNAs can serve as molecular signals because they always show cell-specific expression and respond to diverse stimuli, indicating that their expression is under a certain degree of transcriptional control. For instance, linc-P21, PANDA and Tug1 are transcriptionally activated in response to DNA damage by the direct binding of the tumour-suppressor protein p53 to their promoters [14–16]. Decoys: one of major means by which lncRNAs regulate transcription is to act as molecular decoys, that is, to bind to a protein and titrate it away without exerting any function. In this archetype, lncRNAs act as a ‘molecular sink’ for RNA-binding proteins. For example, the lncRNA, Gas5 (growth-arrest specific 5), can interact with the DNA-binding domain of glucocorticoid receptors (GR), preventing them from binding to DNA response elements [17]. This kind of lncRNA always exerts a negative regulatory role on an effector, so knockdown of these lncRNAs may mimic a gain-of-function of its target protein and a rescue phenotype can be obtained by a double knockdown of both lncRNA and the effector [13]. Guides: lncRNAs bind to a protein and then direct the localization of the ribonucleoprotein complex to specific targets to regulate gene expression, either *in cis* (of neighbouring genes) or *in* trans (of distantly located genes). For example, the lncRNA, HOTAIR, directs the chromatin-modifying complexes, Polycomb Repressive Complex 2 (PRC2) and LSD1, to target genes *in trans* [14,18–20], while, Air, Kcnq1ot1 and Evf-2 target chromatin-modifying complexes to their targets *in cis* [21–24]. Knockdown of this kind of lncRNA may phenocopy loss-of-function of the effector. Different from the decoy archetype, both knockdown of lncRNA and effector leads to an exacerbated phenotype [13]. Scaffolds: in this archetype, lncRNAs possess different domains providing platforms to assemble different effector molecules that function together, thereby precisely controlling the intermolecular interactions and signalling events. For example, HOTAIR can act as a scaffold and bridges between PRC2 and the LSD1/CoREST complex forming the HOTAIR/PRC2/LSD1 complex, which can suppress target gene expression [20]. Knockdown of these lncRNAs can produce a similar effect to that expected from the decoy archetype. Understanding these archetypes is critical in the study of lncRNA functions and for their exploitation to realize the prevention and control of human diseases.

**Fig. 3:**
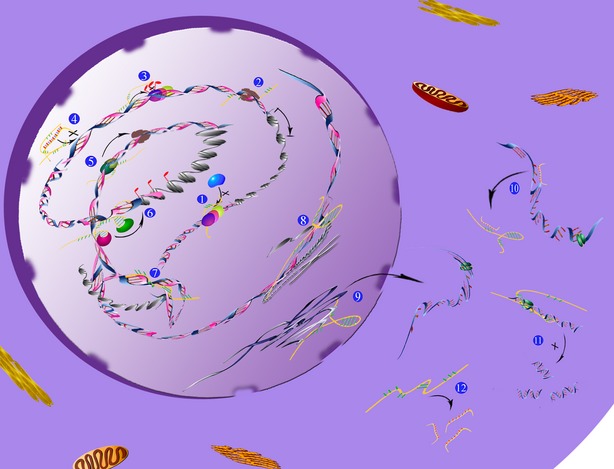
Mechanisms of gene expression regulation by lncRNAs – at three different levels. Transcriptional level: (1) Inhibit the combination of transcription factor and promoter. (2) Regulate gene transcription by interacting with RNA PII. (4) Control gene transcription by functioning as an endogenous competitive RNA. (5) Function as a transcription factor co-activator. (7) Form triple helical structures with DNA. Post-transcriptional level: (9) Regulate the alternative splicing of pre-mRNAs. (10) Influence translation of mRNAs by interacting with miRNAs. 11) Forming double-stranded RNA with mRNA to enhance the stability of mRNA. (12) Cleave into small non-coding RNAs. Epigenetic regulation level: (3) Control DNA methylation (influence methylation of promoter CpG islands). (6) Regulate histone modification (methylation, acetylation and ubiquitination). (8) Combine with chromatin modification complexes. Detailed descriptions are in the main text.

LncRNAs participate in various molecular genetic and cellular processes [25], including chromosomal dosage compensation, control of imprinting, chromatin modification, maintenance of chromatin structure, transcription, splicing, translation, cellular differentiation, integrity of cellular structures, cell cycle regulation, intracellular trafficking, reprogramming of stem cells and the heat shock response. Among all of these functions, regulating gene expression is of great importance in understanding the roles lncRNAs play in tumourigenesis. In contrast to the small ncRNAs, which are highly conserved and participate in transcriptional and post-transcriptional gene silencing, lncRNAs are poorly conserved and regulate gene expression through diverse mechanisms at different levels as follows (Fig. 3):

1. at the transcriptional level: (*i*) lncRNA transcription upstream of a target gene, which can disturb the transcription of the downstream target gene by impeding the association of transcription factor and promoter, thus inhibiting transcription of the target gene; (*ii*) lncRNAs functioning as co-activators with transcription factors by interacting with transcription factors to regulate gene transcription; (*iii*) lncRNAs interacting with DNA forming triple helix structures, thereby influencing target gene transcription; (*iv*) lncRNAs regulating target gene transcription by interacting with RNA PolII; (*v*) lncRNAs controlling target gene transcription by functioning as an endogenous competitive RNA;
2. at the post-transcriptional level: (*i*) some lncRNAs can be cut into small non-coding RNAs that then exert their mRNA regulation functions; (*ii*) some lncRNAs can interact with mRNAs forming double-stranded RNAs, and this enhances the stability of the mRNA, protecting it from degradation; (*iii*) some lncRNAs can regulate the alternative splicing of pre-mRNAs; (*iv*) lncRNAs can influence the translation of mRNAs by interplaying with miRNAs.
3. at the epigenetic regulation level: (*i*) lncRNAs can regulate histone modification, such as methylation, acetylation and ubiquitination; (*ii*) lncRNAs can control DNA methylation, which is important for epigenetic regulation (for example, lncRNAs can influence target gene expression by controlling the methylation level of its promoter region CpG island); (*iii*) about 20% of intergenic lncRNAs can combine with chromatin modification complexes that regulate chromatin remodelling, gene expression and tumourigenesis.

Although only a small number of functional lncRNAs have been reported to date, lncRNAs have been shown to have important roles in physiological and pathological processes in various kinds of tumours (Table 1). With the emergence of lncRNAs, a new field of molecular biology is growing. Learning the mechanisms of lncRNAs' involvement in tumourigenesis will lead to a new direction in cancer diagnostics and treatment.

**Table 1:** Cancer-related lncRNAs

LncRNA	Tumour type	Mechanisms	Potential application	Reference
AK023948	PTC	Down-regulated	A candidate gene for PTC predisposition	[26]
AK126698	NSCLC	Decreases NKD and increases the accumulation and nuclear translocation of β-catenin	A potential molecular target for reversing NSCLC cisplatin resistance	[27]
ANRIL/p15AS	Prostate, leukaemia	Activates PRC1 and PRC2, suppressing p15^INK4b^/p14^ARF^/P1 6^INK4a^	A molecular mechanism underlying epigenetic transcriptional repression	[28,29]
AFAP1-AS1	BE, EAC	Hypomethylated	A potential therapeutic target	[5,6]
BC200	Lung, cervix, oesophagus, breast, ovary, parotid, tongue	Up-regulated	A molecular tool in the diagnosis and /or prognosis of breast cancer	[6,7]
CTBP1-AS	Prostate	Represses CTBP1 expression by recruiting PSF along with histone deacetylases; promoting cell cycle by inhibiting tumour-suppressor genes *via* the PSF-dependent mechanism	Proto-oncogenic and tumour-suppressive effects	[8]
GAS5	Breast, prostate	Induces growth arrest and apoptosis; prevents GR-induced gene expression	Acting as a tumour suppressor	[9,10]
H19	Bladder	Promotes proliferation by regulating ID2 expression	Providing a platform for developing an effective treatment strategy for bladder cancer	[30]
HOTAIR	Lung, breast, liver, colon, pancreas, oesophagus	Recruits PRC2 and/or lysine-specific chromatin loci	A potential biomarker for lymph node metastasis in HCC; A molecular marker in EC; A potential chemotherapy targeting	[31]
HULC	HCC	Up-regulated	A plasma biomarker for detecting HCC	[32,33]
Loc258194	Osteosarcoma	Down-regulated	Tumour-suppressor lncRNA; prognostic biomarker	[34]
LncRNA-LET	Lung, liver, colorectal	Repressed by hypoxia-induced histone deacetylase 3	Provide avenues for therapeutic agents against cancer progression	[35]
lncRNA-DQ786227	Lung	Up-regulated	Provide new insight into the underlying mechanisms of chemical carcinogenesis	[36]
MALAT1/NEAT2	NSCLC, prostate, colon, liver, uterus	Up-regulated	A prognostic marker for HCC following liver transplantation; A diagnostic and prognostic biomarker in NSCLC; A potential therapeutic target for castration-resistant prostate cancer	[37–40]
MEG3	Prostate, lung	Induces apoptosis through p53 signalling; down-regulation	Functioning as a tumour suppressor and a potential therapeutic target against NSCLC	[41–43]
PCA3/DD3	Prostate	Up-regulated	A unique diagnostic biomarker for PCa	[41]
PCAT-1	Prostate	Inhibits BRCA2 and promotes cell proliferation	A potential therapeutic target	[44]
PVT1	Medulloblastoma multiple myeloma	Cmyc-pvt1 fusion protein	The first recurrent translocation reported in medulloblastoma	[45]
Spry4-it1	Melanoma	Up-regulated	Playing an important role in the molecular aetiology of human melanoma	[46]
SRA	Breast, uterus, ovary	Regulates gene expression mediated by steroid receptors	A potential biomarker of steroid-dependent tumours	[47]
TUC338	HCC	Promotes cell proliferation	A potential therapeutic target for HCC	[48]
UCA1/CUDR	Lung, bladder, colon, cervix, lung, thyroid, liver, breast, oesophagus, stomach	Up-regulated	A promising biomarker for bladder cancer invasion and progression; A potential therapeutic target in bladder cancer	[49]

PTC, Papillary thyroid carcinoma; BE, Barrett's oesophagus; EAC, oesophageal adenocarcinoma; EC, endometrial carcinoma; HCC, hepatocellular carcinoma.

## Expression of LncRNAs in NSCLC

**Fig. 4:**
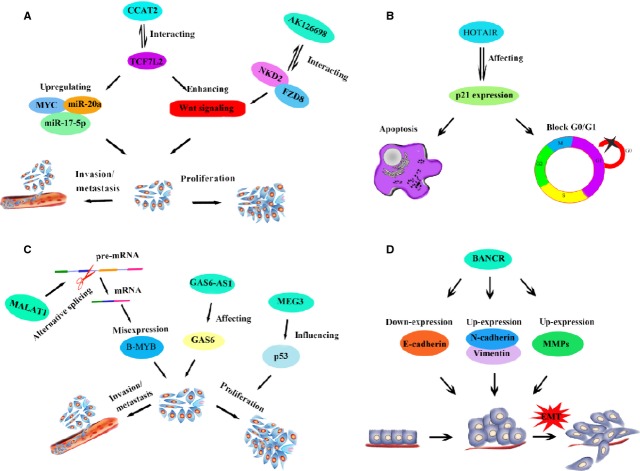
Schematic plot of lncRNAs expressed in NSCLCs and their functions. (**A**) The function pathways of two lncRNAs: CCAT2 and AK126698; (**B**) The function pathway of HOTAIR; (**C**) The function pathways of three lncRNAs: MALAT1, GAS6-AS1 and MEG3; (**D**) The function pathway of BANCR. Detailed descriptions are in the main text.

LncRNAs have multiple functions in tumourigenesis; hence, identification of cancer-associated lncRNAs and investigation of their biological functions and molecular mechanisms are important for understanding the development and progression of cancer. Accumulating evidence shows that lncRNAs participate in the progression of NSCLC. The study of tumour-suppressor lncRNAs provides a new avenue for understanding the pathogenesis and development of NSCLC, and provides a new platform for seeking more efficient therapeutic agents against NSCLC. Here, we introduce several misexpressed lncRNAs (Fig. 4) that are associated with NSCLC and discuss their potential clinical application in diagnostics, prognostics and treatment.

## Onco-lncRNAs

The development and progression of tumours is precisely regulated by numerous biological processes, which often involve silencing tumour-suppressor genes or activating oncogenes. The initiation of NSCLC is no exception. An oncogene is defined as a gene whose product can promote tumour initiation and progression. Activation of oncogenes plays a significant role in the molecular pathogenesis of human tumours. Therefore, finding new oncogenes and investigating their functions is essential for developing new therapeutic targets. As for translated genes, lncRNAs are also divided into onco-lncRNAs and tumour-suppressor lncRNAs. Metastasis-associated lung adenocarcinoma transcript 1 (MALAT1), CCAT2, HOTAIR and AK126698 are onco-lncRNAs and their overexpression can facilitate the progression of NSCLC by promoting cell growth, migration and invasion.

## MALAT1

Owing to limited diagnostic technology and lack of cancer awareness, NSCLC is usually diagnosed at an advance stage, resulting in an unsatisfactory 5-year survival rate. To improve the survival rate, effective diagnostic and prognostic biomarkers urgently need to be found. As lncRNAs have multiple functions in tumour progression [50], research into lncRNAs as biomarkers has increased. Presently, only a few lncRNAs have been demonstrated as biomarker candidates in patients' body fluids [51], such as HULC (highly up-regulated in liver cancer), which is expressed at high levels in hepatocellular carcinoma [32]. prostate cancer gene 3 (PCA3) can be detected with high accuracy in the urine of prostate cancer patients [52]. MALAT1 can be used as a candidate blood-based biomarker for the diagnosis of NSCLC, while MALAT1 in paraffin-embedded tissues can indicate a poor prognosis in NSCLC and induces migration and tumour growth [37,39,53].

MALAT1, also known as nuclear-enriched transcript 2 (NEAT2), is a highly conserved lncRNA with an 8.7 kb transcript. It is located on chromosome 11q13 and is a highly abundant nucleus-restricted RNA that localizes to nuclear speckles. In human cells, MALAT1 can interact with several pre-mRNA splicing factors, including serine arginine dipeptide-containing SR family splicing factors, to influence alternative splicing of pre-mRNAs and modulate the cellular distribution and activity of SR splicing factors. Tripathi *et al*. proposed that aberrant expression of MALAT1 in different cell types and cell cycle phases acts as a ‘molecular sponge’ titrating the cellular pool of SR splicing factors [54].

Overexpression of MALAT1 has been shown in various tumours, such as breast, prostate, colon and liver, as well as NSCLC, especially in early-stage metastasizing patients [40,55–57]. Ping *et al*. have reported that MALAT1 can predict metastasis in early-stage NSCLC [53]. They found that expression of MALAT1 was significantly associated with metastasis in a large cohort of NSCLC patients. Consistent with Ping *et al*., Lars *et al*. verified that MALAT1 stimulates migration, invasion and tumour growth, [37], although the underlying mechanism is poorly understood. One possible explanation is that aberrant expression of MALAT1 in specific cells leads to abnormal alternative splicing, resulting in misexpression of genes, such as the oncogenic transcription factor, B-MYB [54]. Together, these findings suggest that MALAT1 participates in the progression of NSCLC and that overexpression of MALAT1 can be used as an indicator to identify whether the tumour has the potential for metastasis. MALAT1 is the first lncRNA shown to regulate NSCLC metastasis.

MALAT1 has recently been shown to be a diagnostic biomarker of NSCLC. Daniel *et al*. detected the MALAT1 expression level in the peripheral blood of 45 NSCLC patients (who had not received treatment) and 25 cancer-free subjects [39]. They found that MALAT1 could be easily detected in NSCLC patients and that this marker fulfilled many of the main characteristics of a diagnostic biomarker: easily accessible, minimal invasion to obtain sample and high specificity. Therefore, they suggested that MALAT1 could be used as diagnostic biomarker for NSCLC. However, there were two drawbacks. First, sensitivity of MALAT1 was not satisfactory; therefore, it cannot be a single marker but a complementary marker. Second, the expression levels of MALAT1 between cancer patients and cancer-free subjects were statistically different; however, the difference between adenocarcinoma (AdCa) and squamous cell carcinoma (SCC) was not significant, indicating that the expression level of MALAT1 has little diagnostic value for discriminating between AdCa and SCC.

In addition to being a diagnostic biomarker, MALAT1 can also indicate poor prognosis in NSCLC patients. Schmidt *et al*. verified that MALAT1 along with thymosin β_4_ can be utilized as independent prognostic parameters for survival in patients diagnosed with early-stage NSCLC [37]. Subsequently, Lars *et al*. demonstrated that overexpression of MALAT1 indicated a poor prognosis in NSCLC patients [37].

## CCAT2

Colon cancer-associated transcript 2 (CCAT2), a novel lncRNA transcript encompassing the rs6983267 single nucleotide polymorphism, is located on 8q24. CCAT2 has been reported to be overexpressed in colorectal and breast cancer [58,59]. Recently, Qiu *et al*. have shown that CCAT2 was highly expressed in NSCLC, especially in LAD and silencing CCAT2 by siRNA could inhibit the proliferation and invasion of NSCLC cell lines *in vitro* [60]. As to the underlying mechanism, Qiu *et al*. did not give an explanation. However, Ling *et al*. have demonstrated that CCAT2 can up-regulate MYC, miR-17-5p and miR-20a through TCF7L2-mediated transcription and knockdown of miR-17-5p or miR-20a in CCAT2-overexpressing cells can lead to a significant decrease in cell migration. Furthermore, crosstalk between CCAT2 and TCF7L2 can result in an enhancement of Wnt signalling activity [58]. We therefore suggest that CCAT2 may function through the same pathway in NSCLC. Elucidation of the precise mechanism requires further study.

Given that CCAT2 is highly expressed in NSCLC, it has the potential to be a diagnostic biomarker. CCAT2 is different from MALAT1 in that it is specifically overexpressed in LAD, while MALAT1 is highly expressed in both LAD and SCC. Therefore, CCAT2 provides a new biomarker to aid the identification of LAD and SCC. Also, Qiu *et al*. have demonstrated that CCAT2 together with serum tumour biomarker carcino-embryonic antigen can significantly increased predictive efficiency, and the combination could predict lymph node metastasis in NSCLC. However, CCAT2 alone as a biomarker for lymph node metastasis was not verified by Qiu *et al*. because the expression level of CCAT2 in NSCLC-negative lymph node was not significant.

## HOTAIR and AK126698

For NSCLC patients, surgery is usually the first choice treatment; however, because diagnosis is usually at a late stage many patients lose the best time for surgery. Therefore, chemotherapy plays a significant role in treating NSCLC. Currently, platinum based combination chemotherapy is the first-line chemotherapy regimen for NSCLC. Among all the platinum based drugs, cisplatin is the most widely used. However, many patients are insensitive to cisplatin, which seriously hampers the efficiency of chemotherapy. The underlying mechanisms are still not completely understood; therefore, study of the chemoresistance mechanism is essential for getting a better chemotherapy response.

HOTAIR, an lncRNA with a length of 2.2 kb [61], is located in the HOXC locus and is transcribed in the antisense orientation [18]. It was the first lncRNA discovered to be involved in tumourigenesis. It facilitates the progression of various tumours by recruiting PRC2 or reorganizing chromatin [19]. In addition, HOTAIR also participates in the chemoresistance to cisplatin in NSCLC. Recently, we investigated the correlation of HOTAIR overexpression with the sensitivity of LAD cells to cisplatin [62]. *In vitro*, we found that HOTAIR was highly expressed in cisplatin-resistant A549/DDP cells compared with parental A549 cells, and knockdown of HOTAIR can restore the sensitivity of A549/DDP cells to cisplatin. *In vivo*, the expression of HOTAIR was down-regulated apparently in cisplatin-sensitive LAD tissues. It has been reported that HOTAIR can interact with PRC and LSD1/CoREST/REST, leading to the modification of DNA-binding proteins and, therefore, regulates gene expression. Epigenetic silencing is a common mechanism to inactivate tumour-suppressor genes. Enhancer of Zeste 2 (EZH2) is a component of the PRC2 that can mediate transcriptional repression by histone methylation. p21, a cyclin-dependent kinase inhibitor induced by p53 after DNA damage or by p53 overexpression, is a downstream target of HOTAIR. Cao *et al*. have shown that p21 levels can be significantly increased in NSCLC cells after EZH2-siRNA delivery, indicating that HOTAIR might regulate the expression of p21 by working in cooperation with the PRC2 [63]. Thus, the potential mechanism of HOTAIR in mediating cisplatin resistance of LAD might be associated with enhancement of apoptosis and G_0_/G_1_ cell cycle arrest by affecting p21 expression.

Additionally, the lncRNA, AK126698, has also been verified to be associated with cisplatin resistance in NSCLC. AK126698, with a length of 3826 bp, exists in the cerebellum and is defined as human cDNA FLJ44744 fis [64]. Yang *et al*. demonstrated that AK126698 may play a significant role in cisplatin resistance of NSCLC. They found the expression level of AK126698 was associated with various members of the Wnt signalling pathway, such as NKD2 and FZD8 [27]. Overexpression of β-catenin not only contributes to the tumourigenesis of NSCLC, but also increases chemoresistance [65]. Meanwhile, NKD2 can inhibit β-catenin by binding to DVL [66]. And knockdown of AK126698 in A549 cells reduced the expression of NKD2 and increased the expression of β-catenin. Therefore, AK126698 can regulate the resistance of cisplatin in NSCLC partially through the Wnt signalling pathway. Overexpression of AK126698 increases the sensitivity of NSCLC to cisplatin.

Both HOTAIR and AK126698 participate in resistance to cisplatin in NSCLC; therefore, they are potential therapeutic targets. As they exert opposite effects (overexpression of HOTAIR contributes to cisplatin resistance, while decreased AK126698 expression increases resistance to cisplatin), opposite approaches should be used to impede their functions. For HOTAIR, employing effective disruptors to down-regulate its expression may increase the sensitivity of NSCLC patients to cisplatin, and sensitizing AK126698 may be an efficient therapeutic intervention to alleviate cisplatin resistance in NSCLC patients. However, the exact mechanism by which HOTAIR and AK126698 regulate chemoresistance need to be further elucidated.

## Tumour-suppressor lncRNAs

Tumour-suppressor genes are defined as genes whose products can inhibit the initiation and progression of tumours. Inactivation of tumour-suppressor genes plays a significant role in the molecular pathogenesis of human tumours. Thus, finding new tumour-suppressor genes and investigating their functions is a critical step for further understanding the mechanism underlying human tumour initiation, and is essential for developing new therapeutic targets. Maternally expressed gene 3 (MEG3), GAS6-AS1 (growth-arrest-specific gene 6 antisense 1 RNA) and BANCR (BRAF activated non-coding RNA) are tumour-suppressor lncRNAs [42], whose down-regulation may promote the development of NSCLC. Studying these lnRNAs provides a new area for understanding the molecular biology of NSCLC metastasis and progression.

## MEG3

Maternally expressed gene 3, an imprinted human gene located on chromosome 14q32.3, is expressed in various normal tissues [67]. Loss of MEG3 expression has been found in many tumours, and re-expression of MEG3 can inhibit tumour proliferation *in vitro* [68–70]. The mechanism of losing MEG3 expression includes gene deletion, promoter hypermethylation and hypermethylation of the intergenic differentially methylated region [42]. The loss of MEG3 expression contributes to the progression of various tumours, including NSCLC.

Recently, Lu *et al*. found that the expression of MEG3 was significantly decreased in NSCLC tissues compared with normal tissues, especially in later stage tumours and in tumours undergoing size increase [43]. Also, overexpression of MEG3 can down-regulate NSCLC cell proliferation and induce apoptosis *in vitro* and arrest tumourigenesis *in vivo*. Furthermore, the overall survival time of patients with normal or strong expression of MEG3 was higher than that of patients with lower expression levels of MEG3. Decreased expression of MEG3 results in low levels of p53 protein. p53, a transcription factor, regulates the expression of various genes resulting in the inhibition of tumour progression. Once p53 is mutated or expressed at a low level, the development of tumours is promoted. Taken together, MEG3 may play a critical role in the development of NSCLC. Consistent with Lu *et al*., another two research groups have also shown that MEG3 could function as a tumour suppressor by inducing the activation of p53 [69,70]. Lu *et al*. have also demonstrated that MEG3 can be utilized as a negative prognostic factor for NSCLC patients and as an indicator of poor survival rate.

## GAS6-AS1

lncRNA, GAS6-AS1 [growth-arrest-specific gene 6 (GAS6) antisense RNA 1], another tumour-suppressor lncRNA, is located at 13q34 and is transcribed in the antisense direction relative to GAS6. The mechanism of losing GAS6-AS1 expression has not been clarified; however, down-regulation of GAS6-AS1 might contribute to the progression of various cancers, including NSCLC. Han *et al*. reported that the expression of GAS6-AS1 was significantly down-regulated in NSCLC tissues compared with adjacent normal tissues and decreased GAS6-AS1 expression was negatively correlated with lymph node metastasis and advanced stages of tumour-node metastasis [71]. The loss of GAS6-AS1 expression might be involved in the development and progression of NSCLC. The underlying mechanism has not been fully deciphered, but it may involve influencing its host gene, GAS6. GAS6, a ligand of the Axl/Sky tyrosine kinase family, was originally identified as a gene induced in growth-arrested cells. Axl is overexpressed, mitogenic and has prosurvival functions in various tumours. It is also capable of mediating proliferation, migration and invasion of glioma cells [72]. GAS6 has the strongest affinity to Axl, and GAS6/Axl is required for migration and invasion in many cancers. The precise molecular mechanisms of GAS6-mediated cell migration and invasion have not been thoroughly studied. One possible explanation is that GAS6 can induce the expression of SLUG in JNK- and ERK1/2-dependent mechanisms *via* the AP-1 activator protein-1 transcription factors, c-Jun and ATF-2. This results in E-cadherin reduction/vimentin induction and cell migration [73]. GAS6-AS1 is on the downstream side of GAS6, and in the study of Han *et al*., they found that GAS6-AS1 levels are inversely correlated with GAS6 mRNA levels. They also investigated the correlation between GAS6 mRNA levels and clinicopathological variables in patients with NSCLC, and found that increased GAS6 levels were positively associated with lymph node metastasis and advanced tumour-node-metastasis (TNM) stage, and predicted a poor prognosis, suggesting that the function of GAS6-AS1 might be mediated by GAS6. Further study is warranted to clarify the underlying molecular mechanisms.

As the down-regulation of GAS6-AS1 is associated with NSCLC, especially in patients diagnosed at late stages, it might be a potential diagnostic target in patients with NSCLC, particularly those with metastasis. In the study reported by Han *et al*., univariate and multivariate analyses also showed GAS6-AS1 expression was an independent predictor for overall survival of patients diagnosed with NSCLC.

## BANCR

BANCR (BRAF activated non-coding RNA) functions as a tumour-suppressor lncRNA and can regulate cell proliferation by mediating cell-growth arrest and inhibiting cell invasion, thereby reducing the incidence of malignancy. It is located on chromosome 9 and is 693 bp. It was first found in melanoma cells by Flockhart *et al*.[74]. It may function in mediating melanoma cell migration. In addition to melanoma, BANCR has also been reported to contribute to the progression of NSCLC.

Recently, Sun *et al*. have reported that BANCR expression was significantly decreased in NSCLC tumour tissues compared with normal lung tissues, and the aberrant expression of BANCR was associated with patients' overall survival time: patients with lower BANCR expression levels had significantly shorter survival times compared with those with higher levels [75]. Sun *et al*. also showed that up-regulation of BANCR expression inhibited of cell viability, migration and invasion, while knockdown of BANCR expression facilitated cell migration and invasion.

The molecular mechanism by which BANCR suppresses invasion and metastasis of NSCLC has not been thoroughly studied, but may involve epithelial-mesenchymal transition (EMT). The main characteristics of EMT are the aberrant expression of N-cadherin and Vimentin and down-regulation or loss of expression of E-cadherin [76–78]. In accordance with the main hallmarks of EMT, loss of BANCR expression can reduce E-cadherin expression, and induce N-cadherin, Vimentin and MMPs (matrix metalloproteases). MMPs also participate in the process of cell migration, indicating that EMT might be one mechanism by which BANCR mediates the invasion and metastasis of NSCLC. Down-regulation of BANCR is associated with larger tumour size, advanced pathology, metastasis distance and shorter overall survival time of patients diagnosed with NSCLC [75]. Thus, aberrant expression of BANCR provides a significant predictive value for TNM staging and it has the potential of being a diagnostic and prognostic biomarker. Metastasis has always been the main cause of death in patients diagnosed with NSCLC. Studying the mechanisms and molecular pathways involved in metastasis is critical for understanding the molecular biology of NSCLC development and progression. The study of BANCR highlights a new perspective in understanding the pathogenesis of NSCLC and contributes to the development of lncRNA-mediated therapeutics.

## Conclusions and future directions

Lung cancer has one of the highest morbidity and mortality rates of all malignant tumours. Many researchers have explored the pathogenesis of NSCLC; however, the underlying mechanism is still not fully understood and treatments are unsatisfactory. To further investigate the mechanism of NSCLC and to explore new treatment strategies, research has focused on the lncRNAs. LncRNAs have been verified to play multiple roles in various pathophysiological processes. LncRNAs are divided into onco-lncRNAs and tumour-suppressor lncRNAs. Whether onco-lncRNAs or tumour-suppressor lncRNAs, aberrant expression of both participates in carcinogenesis and they represent significant untapped biomarkers for the diagnosis and prognosis of NSCLC, as well as potential targets of lncRNA-mediated therapy.

Currently, the main cause for poor therapeutic efficacy is late stage diagnosis and chemoresistance; therefore, identifying biomarkers with high sensitivity and specificity and determining the mechanism of chemoresistance is an urgent problem to be solved. In this review, we introduced seven lncRNAs and their roles in NSCLC. Among these lncRNAs, some can be used as biomarkers and some are potential therapeutic targets. However, none of these lncRNAs have been exhaustively studied, and, to date, none are clinically involved in diagnosis, prognosis or treatment.

Numerous lncRNAs are misregulated in NSCLC; however, few have been associated only with NSCLC. The diagnostic rate is likely to be improved by a combination of different lncRNAs and a critical goal is to identify many more lncRNAs that could potentially serve as biomarkers for specific disease states. LncRNAs are also crucial to a full understanding of the chemoresistance mechanism and many are likely to represent therapeutic targets. The actions of lncRNAs will involve many signalling transduction pathways. Study of these pathways may yield critical signalling intersections that could be blocked to impede tumour progression. Of course, these assertions require much focused study to yield clinically relevant findings.

The aberrant expression of lncRNAs in cancer gives rise to the question of how structural variations in lncRNA genes, such as gene amplifications, deletions and base mutations, may favour cancer predisposition. Accumulating evidence has shown that even a small mutation in an lncRNA can influence the lncRNA's structure and thus affect its regulatory functions, which may result in disease, including tumourigenesis. Therefore, further research is needed to elucidate the mechanisms affected by lncRNAs mutations. The potential applications of lncRNAs in biotechnology and medicine are tremendous. Several lines of evidence have verified that lncRNAs are useful as novel diagnostic and prognostic biomarkers and are therapeutic targets in many kinds of cancers, although such clinical applications still require intensive studies before they can be applied. As for NSCLC, lncRNAs are very promising as markers in early-stage patients and may become particularly useful in non-invasive screening. In addition, lncRNAs may prove useful as predictive markers for chemotherapy sensitivity, leading to better treatment tailoring. Finally, the emerging roles of lncRNAs in the progression of NSCLC lay a good foundation for further study and provide a new train of thought for developing more efficient therapeutic agents.
